# Are uk higher psychiatry trainees using special interest sessions to develop their career pathways as intended? what might help achieve this goal?

**DOI:** 10.1192/j.eurpsy.2021.1586

**Published:** 2021-08-13

**Authors:** F. Clay

**Affiliations:** Old Age Psychiatry, Fulbourn Hospital, Cambridge, United Kingdom

**Keywords:** special interest sessions, career pathway, training

## Abstract

**Introduction:**

UK Psychiatry Trainees are allocated one day per week in their final three years of training to use for “a clinical or clinically related area of service which cannot be provided within the training post but which is of direct relevance to the prospective career pathway of the trainee”. It is unclear how trainees in the East of England are using this time and what could help them optimise use of this time. We completed a survey to evaluate these areas.

**Objectives:**

To determine details of how Special interest sessions (SIS) are spent by trainees: How much support/ planning for SIS is available and if this is adequate. Whether trainees feel they are able to use their SIS for its intended purpose of providing “a clinical or clinically related area of service which cannot be provided within the training post but which is of direct relevance to the prospective career pathway of the trainee” Exploration of barriers/tensions to maximizing use of SIS. SIS Record keeping What advice would trainees give re: special interest sessions to a new SPR? What lessons can be drawn to assist trainees from other countries/ training programmes to maximise their own development.

**Methods:**

Survey sent to all Higher trainees in the East of England via Regional Training Programme.

**Results:**

Awaited. Survey sent 29/09/2020

**Conclusions:**

Results pending. We will feedback in detail on outcomes from the survey and subsequent discussion with Regional training programme members.

